# Tailored internet-administered treatment of anxiety disorders for primary care patients: study protocol for a randomised controlled trial

**DOI:** 10.1186/1745-6215-13-16

**Published:** 2012-02-09

**Authors:** Lise Bergman Nordgren, Gerhard Andersson, Åsa Kadowaki, Per Carlbring

**Affiliations:** 1 Department of Behavioural Sciences and Learning, Linköping University, 581 83 Linköping, Sweden; 2 Department of Clinical Neuroscience, Psychiatry Section, Karolinska Institutet, 171 76 Stockholm, Sweden; 3 Swedish Institute for Disability Research, Linköping University, 581 83 Linköping, Sweden; 4 County council of Östergötland, 581 91 Linköping, Sweden; 5 Department of Psychology, Umeå University, 901 87 Umeå, Sweden

**Keywords:** Primary care, anxiety, depression, co-morbidity, Internet-administered cognitive behaviour therapy, cognitive behaviour therapy

## Abstract

**Background:**

Internet-administered cognitive behavioural therapy (ICBT) has been found to be effective for a range of anxiety disorders. However, most studies have focused on one specific primary diagnosis and co-morbidity has not been considered. In primary care settings, patients with anxiety often suffer from more than one psychiatric condition, making it difficult to disseminate ICBT for specific conditions. The aim of this study will be to investigate if ICBT tailored according to symptom profile can be a feasible treatment for primary care patients with anxiety disorders. It is a randomised controlled trial aimed to evaluate the treatment against an active control group.

**Methods:**

Participants with anxiety disorders and co-morbid conditions (N = 128), will be recruited from a primary care population. The Clinical Outcome in Routine Evaluation (CORE-OM) will serve as the primary outcome measure. Secondary measures include self-reported depression, anxiety, quality of life and loss of production and the use of health care. All assessments will be collected via the Internet and measure points will be baseline, post treatment and 12 months post treatment.

**Discussion:**

This trial will add to the body of knowledge on the effectiveness of ICBT for anxiety disorders in primary care. The trial will also add knowledge on the long term effects of ICBT when delivered for regular clinic patients

**Trial Registration:**

ClinicalTrials.gov: NCT01390168

## Background

Anxiety symptoms are one of the most common complaints in mental health care [1]. In Europe, the 12-month prevalence for any anxiety disorder is nearly 14 per cent [2,3]. When broadening the term to mental disorder, the number of people affected in the European Union is 23.5 per cent [3], and co-morbidity rates are high. The most common co-morbid disorder for people with one anxiety disorder is mood disorders and other anxiety disorders [4]. Although the number of people suffering from mental disorders is high, only a small number receive treatment [5], and a large number of adult patients with anxiety disorders are treated in primary care settings and not in specialised care [6].

In an attempt to lower the barriers for help, Internet-based cognitive behavioural therapy (ICBT) has been shown to be effective [7]. ICBT can be, defined as:

"A therapy that is based on self-help books, guided by an identified therapist, which gives feedback and answers to questions, with a scheduling that mirrors face-to-face treatment, and which can also include interactive online features such as queries to obtain passwords in order to get access to treatment modules" ([7] p. 164).

This approach is different from pure self-help, in which no feedback is given from the clinician. Including guidance has been shown to be important in order to optimise outcomes [8-10]. ICBT has shown promising results for a range of somatic and psychiatric conditions. Research has shown similar outcomes as in face-to-face therapy [11]. Moreover, there are several meta-analysis showing promising results for anxiety disorders as well as depression [10,12-14]. However, Coull and Morris [15] found that even if guided self-help seems to have a positive immediate effect, effectiveness at long-term follow-up is considerably diminished. Furthermore, the effects among clinically representative samples are largely unknown. In addition, most studies only target one specific condition, leading to high exclusion rates, especially in media-recruited samples. If participants with co-morbid disorders are included, the co-morbidity is generally not targeted, since the Internet treatment usually has manualised protocols leading to limited opportunities for participants and therapists to influence the treatment course. In face-to-face treatment, a behaviour analysis or a case conceptualisation is the starting point, and a treatment plan is designed around the information collected from the face-to-face appointment, by carrying out tailored treatments, these problems are addressed.

There is some evidence that tailored ICBT is a feasible option in a heterogeneous self-recruited group of anxiety participants [16]. In that study long-term treatment benefits were found. There is some additional evidence indicating that the effects of guided ICBT are maintained over longer time periods [17-19].

This study will consist of a sample referred via primary care contacts with the aim to test tailored ICBT in an effectiveness study.

## Methods/Design

### Design

We will conduct a randomised controlled trial with two conditions: (1) ICBT with scheduled guidance and (2) an active waiting list control group: "attention control". The study has been approved by the regional Ethical Board and has been registered in clinicaltrials.gov (NCT01390168).

### Study population

The study population will consist of adult primary care patients fulfilling the DSM-IV-TR [20] criteria of any specific anxiety disorder, or anxiety disorder not otherwise specified. Hence, all participants must have clinically significant symptoms. Co-morbidity will not serve as a reason for exclusion, with the exception of health conditions that will hinder participation in the study and a few additional exclusion criteria. Participants will be included regardless of co-occurring depression; however, the anxiety disorder will have to be the primary diagnosis and complaint. Inclusion criteria will be the following: the person must be 18 years of age or older; they must have Internet access; they must be able to take part in a telephone-administered diagnosis interview; they should not be receiving any ongoing psychological treatment; and they should not be suffering from clinical or sub-clinical anxiety symptoms, if the patient is undergoing any medical treatment for their condition, the dosage has to be stable for at least three months prior to the start of the treatment. Participants with a suicidal risk, defined as scoring 4 or higher on item 9 of the Montgomery Åsberg Rating Scale-Self-rated (MADRS-S [21]), and who are judged to show a high risk in the SAD PERSONS [22] interview will be excluded. Participants with ongoing alcohol dependence will be excluded as well.

### Sample size

Power calculations based on a moderate effect size of *d *= 0.50, comparing the Clinical Outcomes in Routine Evaluation-Outcome Measure (CORE-OM; [23]) scores of the intervention group with the control group with a two-sided t-test (alpha .05, power 80%). This is in accordance with Chambless and Hollon's [24] recommendations for empirically supported therapies. Hence, the N will be set at 128 participants, with 64 in each arm.

### Recruitment

Participants will be recruited from a clinical population-typically by their physician when seeking help at their primary care setting. Eligible participants will be given an information folder containing brief information about the trial and the link to the study website. On the website they will find further information, an informed consent form, a link to the questionnaires serving as screening and the application for participation. The informed consent form will be sent by surface post.

### Randomisation and procedure

Initial selection will take place via computerised screening, consisting of the CORE-OM; [23], the self-rated version of the MADRS-S [25], Beck Anxiety Inventory (BAI;[26]), Quality of Life Inventory (QOLI;[27]), and 10 additional questions regarding demographics and current and past treatment. These results will later be used as a pretreatment assessment.

Participants who fulfil the initial inclusion criteria, according to the computerised screening, will be contacted to take part in a telephone interview where they will be interviewed using the Structured Clinical Interview for DSM-IV-Axis I Disorders (SCID-I; [28]) by seven MSc clinical psychology students trained in using the interview. A psychiatrist and a clinical psychologist will then assess the SCID protocols together with the interviewers. The interviewer, the psychiatrist and the clinical psychologist will subsequently tailor the treatment according to each participant's unique set of problems and to some extent preferences. All participants will be randomised by an online true random-number service independent of the investigators and therapists. The post-treatment assessment will take place 10 weeks after the participants have started their treatment, regardless of how many treatment modules have been completed. There will also be a measurement at 12 months post treatment. All assessments, except the diagnosis interview at pretreatment, are self-reported and will be conducted via the Internet.

All communication will be administered via an online messaging system resembling standard e-mailing systems connected to the study. All data is encrypted in the database and a cryptographic protocol (Secure Sockets Layer) will be used to provide communication security over the Internet.

### Intervention

#### Treatment

The treatment will cover a period of 10 weeks and consist of a maximum of 10 modules, or chapters, per participant. The modules are based on cognitive-behavioural therapy (CBT) and shown to be effective when used on specific diagnosed samples (i.e. panic disorder [29], social phobia [30], GAD [19], depression [31]). Modules will be rewritten to fit together in the tailored format (e.g., words relating to specific conditions will be removed like "panic disorder").

Each module consists of a text (9-39 A4 pages) presenting a specific symptom (e.g. agoraphobia), exercises and is completed by three to eight essay questions to be worked through during a period of one week. Some of the modules (i.e. relaxation and mindfulness) have audio files attached to them for the participants to listen to. The homework questions are intended to encourage learning and to help the Internet therapist assess whether the participants have assimilated the material or not. Participants will be asked to answer the questions and provide worksheets and report on outcomes from different exercises to their therapist and will then be served with individual feedback. When the therapist receives a homework assignment showing that the participant has assimilated the material, the next module will be made accessible through an encrypted message exchange system. If not posted in time, the therapist will send a reminder message, and if the submitted homework does not show adequate knowledge, the therapist will send feedback to the participant regarding how to work on the area further. Prescribed modules are available for download in PDF format and participants will be advised to print out the self-help material to make it easier to take notes and to have the material readily available. The therapists will be told not to spend more than 15 minutes per participant per week in reading and communicating.

The first module (introduction) and the last (relapse prevention) are fixed, which gives the clinician the possibility to tailor the treatment by adding any of the following: cognitive restructuring (2 modules), social anxiety (2 modules), generalised anxiety (3 modules), panic disorder (2 modules), agoraphobia, behavioural activation (2 modules), applied relaxation, mindfulness, assertiveness, problem solving, stress management and sleep.

#### Attention control

Participants in the attention control group are asked questions about their well-being on a weekly basis by their therapist. Each participant will e-mail their answers to the therapist once a week, but they are generally not given any feedback on their answers.

### Instruments

All questionnaires, except the diagnostic interview, will be administered via the Internet. The reason for doing this is to minimise the risk for keying errors and to be cost-effective. Research has shown that Internet-administered self-reporting of these questionnaires is as equally valid as the paper and pencil versions [32,33]. The diagnostic interview (SCID-I) will be conducted by telephone, a format shown to be as equally valid as face-to-face administration [34].

All instruments will be used at pretreatment, post treatment and at 12-month follow-up, with the exception of demographic data and diagnosis interview at post treatment and in the long-term follow-up.

#### Demographic data

Ten background questions will be asked about age, gender, education, past and previous treatment (both psychological and medication) and employment status.

#### Diagnosis

The Structured Clinical Interview for DSM-IV-Axis I disorders (SCID-I; [28]) will be used to investigate the presence of a diagnosis. This is a semi-structured interview to assess for DSM-IV Axis I-diagnoses.

#### Primary outcomes

The CORE-OM [23] is a 34-item scale covering four subscales of subjective well-being. The scales are: symptoms (anxiety, depression, physical problems, trauma), functioning (general functioning, close relationships, social relationships) and risk (to self and others). Items are scored from 0-4 and relate to the preceding week. CORE-OM clinical scores can range from 0-40, with higher scores indicating greater severity. Internal consistency has been reported as *α *= .94 [23].

#### Secondary outcomes

For the measurement of anxiety symptoms, Beck Anxiety Inventory (BAI; [26]) will be used. The BAI contains 21 short questions and scoring varies from 0-63. A score of 30 or higher is considered to correspond to severe anxiety symptoms. Internal consistency is high [26], with Cronbach's alpha ranging from .90 to .94. and test-retest reliability at *r *= .75.

Depressive symptoms will be measured using the MADRS-S [25]. The scale consists of nine items rated on a seven-grade scale measuring depressive symptoms over the past three days. MADRS-S was developed to measure changes in depressive symptoms and was originally a clinical-rated version. The self-rated version has shown a high degree of concordance with the clinical-rated one [35]. Total scoring varies from 0-54, with scores above 35 [36] considered as severe. This scale will also serve as an initial indicator for suicidal risk using the participants scoring on item 9 (suicidal thoughts) as a marker for further investigation of the matter. Concurrent validity is shown to be good with a test-retest reliability of *r *= .83 [37].

As noted earlier, the use of MADRS-S and the scoring on the suicidal thoughts item indicate the necessity for further investigation on suicidality via a telephone-administered interview conducted by a psychiatrist using the SAD PERSONS scale [22]. The SAD PERSONS is an acronym for 10 major risk factors (Sex, Age, Depression, Previous attempt, Ethanol abuse, Rational thinking loss, Social supports lacking, Organised plan, No spouse, Sickness) and is used together with a clinical evaluation carried out by the interviewer for assessing the likelihood of suicidal attempts [22].

For the measurement of quality of life we will use the QOLI [27]. This scale assesses subjective degree of satisfaction in 16 different areas of life (e.g. health and work). The scale show high internal consistency (α = .77-.89), and a test-retest reliability from *r *= .80 to .91 [27].

For the measurement of loss of production and health-care consumption the Trimbos and Institute of Medical Technology Assessment Questionnaire on Costs Associated with Psychiatric Illness (TiC-P; [38]) will be used. We will use part I (direct costs of health-care consumption) and part II (indirect costs of loss of production).

### Analyses

When analysing post-treatment data and data collected at one-year follow-up, an intention-to-treat design will be used, to include all participants, regardless of their compliance, withdrawal or protocol deviations. Between-group changes at post treatment and at one-year-follow-up will be calculated using analyses of covariance (ANCOVAs), with pre-treatment scores as the covariate. This approach is proven to be a reliable strategy in analysing the results of baseline and follow-up data in RCTs [39]. Effect sizes between and within the two groups will be calculated with Cohen's *d *computed with the pooled standard deviation.

## Discussion

The trial described in this protocol is a randomised controlled trial targeting individuals aged 18 years or older who seek their primary care setting for emotional distress. The individuals suitable for this treatment will be randomised into either treatment or an active control group. Outcome measures post treatment will be compared with those of the wait list control group. The primary aim is to assess the effectiveness of the tailored online treatment both post treatment and at a one-year follow-up.

Given the large number of individuals suffering from mental disorders, and the small proportion receiving adequate treatment for their condition, increasing the accessibility of effective treatment is important. Given that the bulk of patients fulfilling an anxiety diagnosis suffer from at least one co-morbid disorder [4] makes it important to investigate if the ICBT format of treatment is applicable to these individuals as well. The main part of earlier studies on ICBT has focused on only one primary diagnosis, leading to high exclusion rates of up to 84 per cent [29]. One of the aims of this study is to investigate whether this kind of treatment could be feasible for primary care patients regardless of anxiety diagnosis and co-morbidity, striving to test the effectiveness of this treatment.

The recruitment of participants to this study is designed to be similar to that of an ordinary process of seeking psychological treatment via a primary care setting. The patient and primary care contact will meet as usual, as they would if they were receiving treatment, and then the potential participant would be recommended to apply for the study. This leaves their primary care contact, in the first instance, to decide which treatment to recommend for the patient's unique set of symptoms. This increases the external validity.

This design has some limitations. One possibility is that the patients selected for the study will be considered being less severe patients-either by the characteristics of their symptoms or by demographic characteristics. Another limitation is the lack of control group in the one-year follow-up. For ethical reasons, the patients in our wait list will be offered the treatment after 10 weeks. In terms of generalisation, it should be taken into consideration that the sample could be selected on other criteria than they would be if they were receiving administered treatments. To reduce this risk, close contact between the primary care settings and the project leader of the study is strived for. Another fact is that the therapists in this study are students in later terms of clinical training, not a clinical psychologist or a counsellor that patients would normally meet in these settings. These issues are partly being addressed by the fact that the students undergo supervision from an experienced clinical psychologist.

## Trial status

The trial is ongoing, still recruiting participants.

## Competing interests

The authors declare that they have no competing interests.

## Authors' contributions

LBN in collaboration with PC, GA and ÅK designed the study. LBN drafted the manuscript. PC and GA reviewed and revised the manuscript. All authors have read and approved the final manuscript to be published.
